# Diffusion Tensor Imaging in Determining Atypical Peripheral Nerve Neuroma in a Patient with a Painless Mass

**DOI:** 10.5152/eurasianjmed.2024.23149

**Published:** 2024-06-01

**Authors:** Fathinul Fikri Ahmad Saad, Razinul Syamim Fathinul Fikri, Ahmad Danial Ahmad Shahrir

**Affiliations:** 1 Centre for Diagnostic Nuclear Imaging, Universiti Putra Malaysia Faculty of Medicine and Health Science, Selangor, Malaysia; 2 Nuclear medicine Unit, Hospital Universiti Putra Malaysia Faculty of Medicine and Health Science, Selangor, Malaysia

To the Editor,

Diffusion tensor imaging (DTI) promises further scrutiny in determining the functional and structural defects of the affected peripheral nerves (PNs) via differences in nerve stiffness and information of early regeneration. The report by Zhang et al^1^ in their study on DTI of axonal and myelin changes in classical trigeminal neuralgia has substantiated that significantly increased fusion anisotropy (FA) is sufficiently sensitive to different pathologic states. In this regard, we emphasize that the reliability of the functional metric of the demyelination in peripheral neuropathy has more favorable credit than conventional diffusion-weighted imaging (DWI) in understanding patients with atypical presentations of the causative factors of peripheral neuropathy. We presented a case of a 71-year-old man who presented with a painless, protracted subcutaneous tissue lump on the medial side of his left forearm, with associated numbness over the first and second web space. The lump was soft and non-tender, with a negative Tinel’s sign on examination. Diffusion tensor imaging scanning protocols were performed on the 3.0 T scanner (Prisma, Siemens Healthineers, Erlangen, Germany), consisting of 13 volumes (45 slices, 128 × 128 voxel, slice thickness 2.2 mm, in-plane voxel size 1.5 mm × 1.5 mm), representing 12 gradient directions and 1 scan with gradient 0 (B0) centered at the left wrist. Echo time (TE) and repetition time (TR) were 93 ms and 8000 ms, respectively. *B* value was 800 s/mm^2^, 5 scans were k-space averaged online by the Siemens SYNGO operating software. A T1-weighted magnetization-prepared rapid-acquisition gradient echo sequence consisting of 160-200 sagittal partitions depending on head size was used. Written informed consent was obtained from the patient who agreed to take part in the study.

The FA values denoting axonal injury showed reducing values, attributed to the biopsy-proven neuroma tissue (Figure 1).^2-5^ On magnetic resonance imaging, the lesion exhibited an intermediate signal on T1W images and water restriction on the DWI images. The DTI image exhibited a lack of regional nerve ramification and associated functional degeneration on the FA parameter (Table 1). Diffusion tensor imaging enhances the functional characterization of nerve regeneration and tissue microstructure and generates parameters on myelination composite, axonal diameter, fiber density, and organization.^6,7^ There is a limited report on the role of DTI in the atypical presentation of neuroma, for which its utility is deemed essential to ascertain accurate diagnosis and would serve as a potent marker in monitoring neuronal repairs post treatment. Its utility has limitations in standard data acquisition on different scanning models. Motion artifacts and soft tissue edema may affect the accurate delineation of its virtual anatomical mapping for surgical planning. In the future, DTI could constantly improve virtual surgical planning and influence precision in medical therapeutics with progress in artificial intelligence on the mathematical algorithms and savvy use of analytical parameters for researchers on personal computers for end users of varied disciplines.

## Figures and Tables

**Figure 1.**
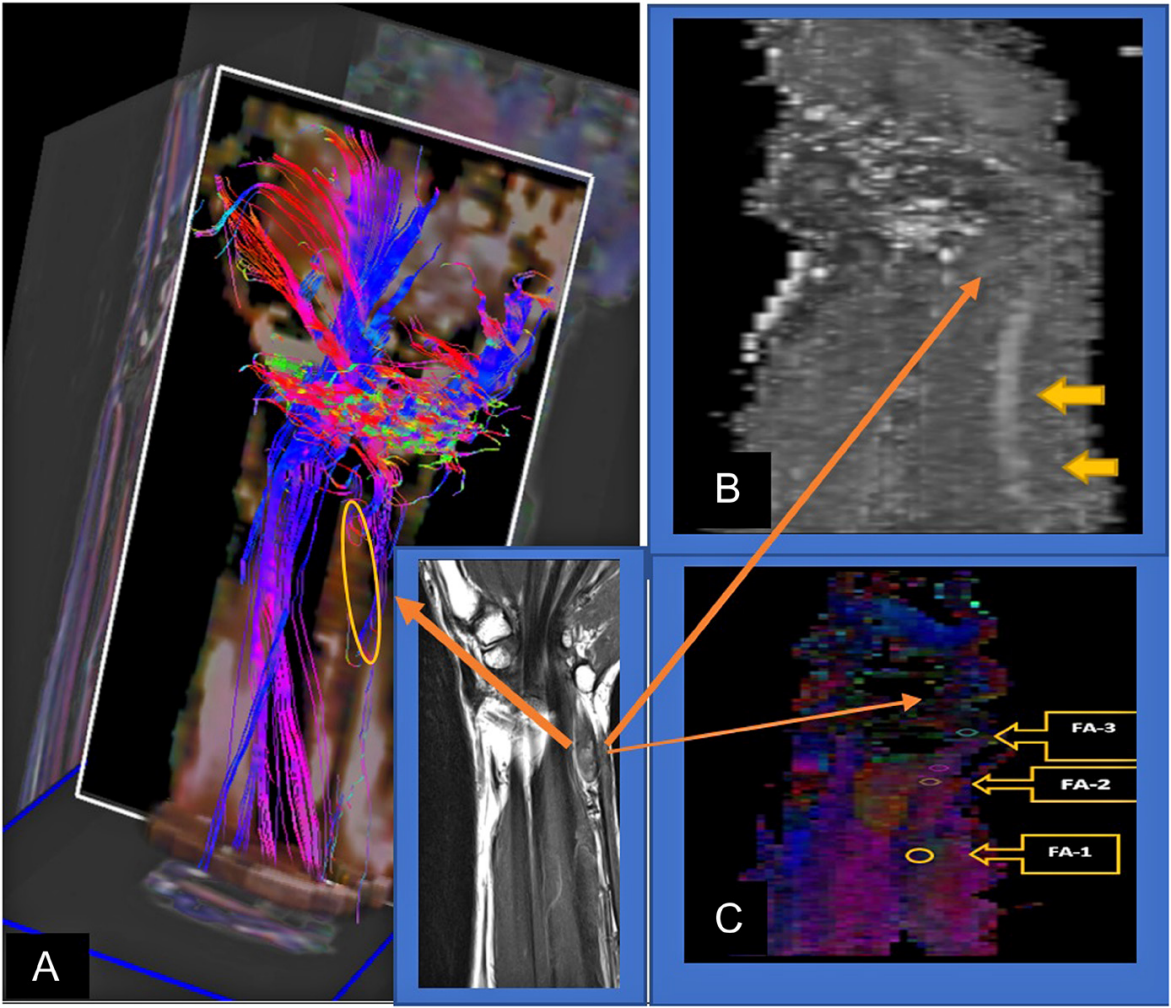
(A) (Inset) T1-weighted reference MR image of the ulnar nerve. Coronal color-coded map shows ulnar nerve (left arrow, eclipse marker). The direction of maximum diffusivity is mapped with triple colors—red, green, and blue color coding. (B) The ADC map confirms the tracked ulnar nerve fiber showing the high signal intensity of the normal caliber (right top arrow). (C) The FA map shows the tracked ulnar nerve fiber with decreasing values of the mean FA values toward the soft tissue lesion at the distal forearm—FA1 (0.96), FA1 (0.86), and FA3 (0.79).

**Table 1.** Reported FA Values of the Normal and Pathological of the Peripheral Nerve Axons

	Nerve Tissue	FA Value (Normal)	FA Value (Abnormal/Pathology)
1	Median nerve	0.58 ± 0.04	0.45 ± 0.05
2	Ulnar nerve	0.48 ± 0.09	0.08 ± 0.14
3	Trigeminal nerve	0.56 ± 0.05	0.43 ± 0.08
4	Tibial nerve	0.45 ± 0.04	0.36 ± 0.04
